# Structural insights into translation regulation by the THF-II riboswitch

**DOI:** 10.1093/nar/gkac1257

**Published:** 2023-01-09

**Authors:** Lilei Xu, Yu Xiao, Jie Zhang, Xianyang Fang

**Affiliations:** Beijing Advanced Innovation Center for Structural Biology, Beijing Frontier Research Center for Biological Structure, School of Life Sciences, Tsinghua University, Beijing 100084, China; Center for Synthetic and Systems Biology, Tsinghua University, Beijing 100084, China; Beijing Advanced Innovation Center for Structural Biology, Beijing Frontier Research Center for Biological Structure, School of Life Sciences, Tsinghua University, Beijing 100084, China; Beijing Advanced Innovation Center for Structural Biology, Beijing Frontier Research Center for Biological Structure, School of Life Sciences, Tsinghua University, Beijing 100084, China; Center for Synthetic and Systems Biology, Tsinghua University, Beijing 100084, China; Beijing Advanced Innovation Center for Structural Biology, Beijing Frontier Research Center for Biological Structure, School of Life Sciences, Tsinghua University, Beijing 100084, China; Center for Synthetic and Systems Biology, Tsinghua University, Beijing 100084, China; Key Laboratory of RNA Biology, Institute of Biophysics, Chinese Academy of Sciences, Beijing 100101, China

## Abstract

In bacteria, expression of folate-related genes is controlled by the tetrahydrofolate (THF) riboswitch in response to specific binding of THF and its derivatives. Recently, a second class of THF riboswitches, named THF-II, was identified in Gram-negative bacteria, which exhibit distinct architecture from the previously characterized THF-I riboswitches found in Gram-positive bacteria. Here, we present the crystal structures of the ligand-bound THF-II riboswitch from *Mesorhizobium loti*. These structures exhibit a long rod-like fold stabilized by continuous base pair and base triplet stacking across two helices of P1 and P2 and their interconnecting ligand-bound binding pocket. The pterin moiety of the ligand docks into the binding pocket by forming hydrogen bonds with two highly conserved pyrimidines in J12 and J21, which resembles the hydrogen-bonding pattern at the ligand-binding site FA_PK_ in the THF-I riboswitch. Using small-angle X-ray scattering and isothermal titration calorimetry, we further characterized the riboswitch in solution and reveal that Mg^2+^ is essential for pre-organization of the binding pocket for efficient ligand binding. RNase H cleavage assay indicates that ligand binding reduces accessibility of the ribosome binding site in the right arm of P1, thus down-regulating the expression of downstream genes. Together, these results provide mechanistic insights into translation regulation by the THF-II riboswitch.

## INTRODUCTION

Tetrahydrofolate (THF) and its derivatives, known as folates, are indispensable elements for normal cellular metabolism in all life forms due to their essential roles in one-carbon transfer reactions (1); however, folate biosynthesis and metabolism in bacteria and mammals are completely different. Many bacteria, along with fungi and plants, can synthesize folates *de novo* and therefore constitute important dietary sources of folates (2,3). In contrast, there is no *de novo* synthesis pathway for mammals to synthesize folate and its analogs (4). As an essential nutrient, folate has to be taken from the diet for humans, and folate deficiency is directly linked to severe human health problems, such as birth defects, cardiovascular diseases, increased cancer risk and so forth (5). Folate biosynthesis and metabolism pathways are therefore attractive targets for the development of therapeutics against diseases caused by bacteria and fungi (6,7), as well as some human diseases including cancers (8–10).

In bacteria, genes associated with many central metabolic pathways are controlled by RNA elements located in the 5′-untranslated regions within their mRNAs, which belong to a class of non-coding RNA called riboswitches (11–13). Riboswitches are generally comprised of two components, an aptamer domain and an expression platform. In response to the binding of ligands, such as essential cellular metabolites, to the aptamer domain, a conformational change occurs that leads to the formation of an alternative structure in the expression platform which carries signals for transcriptional or translational machinery, thus triggering ‘ON’ or ‘OFF’ of downstream gene expression at the transcriptional or translational level (14–16). Currently, >40 classes of riboswitches have been identified as recognizing a variety of ligands (17,18). Among them, riboswitches responsive to protein coenzymes or their immediate precursors or byproducts represent the most abundant and diverse groups (19).

More than a decade ago, a class of THF-sensing riboswitch (THF-I) was found to control genes associated with folate synthesis (*folC* and *folE*) and transport (*folT*) in many Gram-positive Firmicutes (20). Crystal structures of the THF-I riboswitch aptamer in complex with various ligands reveal an ‘inverted’ three-way junction (3WJ) architecture formed between P2, P3 and P4 helices, and further stabilized by a long-range pseudoknot (PK) interaction between the internal loop (J2/1) and the P3 apical loop (L3) (21,22). The apo- and holoaptamer structures reported by Huang *et al.* are nearly identical and only a single ligand-binding site near the 3WJ (site FA_3WJ_) is identified (21). However, the holo-structure reported by Trausch *et al.* reveals two ligand-binding sites with one near the 3WJ (site FA_3WJ_) and the other in the minor groove of the PK (site FA_PK_) that bridges P2 and P3 (22). In both holo-structures, the pterin moiety contributes to the most interactions between the THF-I riboswitch and ligands. Further studies demonstrated that only the site FA_PK_ is essential for gene expression regulation (23). These results provide insights into the regulatory mechanisms of the THF-I riboswitch and may help in the development of RNA-targeting compounds.

Recently, a second class of THF riboswitch, named THF-II, was identified in Gram-negative bacteria (24). The THF-II riboswitches are commonly found upstream of *folE* genes, which encode the enzymes that catalyze the first reaction in the *de novo* folate biosynthesis pathway (24,25). Distinct from the aptamers of the previous THF-I riboswitch found in Gram-positive bacteria, the THF-II riboswitch aptamer adopts a simple architecture in which the secondary structure consists of two helices of P1 and P2, two junctions of J12 and J21, and an apical loop L2. Strikingly, the putative ribosome-binding site (RBS) sequence of the adjacent downstream gene locates in the right arm of P1, suggesting that the THF-II riboswitch aptamer encompasses part of its expression platform (24). In addition to their differences in phylogenetic distribution and aptamer architecture, in-line probing assays suggest that the binding pockets in their aptamers are distinct to discriminate ligand characteristics (24). For example, the THF-II riboswitch rejects THF analogs with a substituent beyond hydrogen at the N5 position of the pterin moiety (24), but N5-modified THF derivatives retain binding affinity for the THF-I riboswitch (21).

To understand the ligand recognition principles and deduce insights into the regulatory mechanisms of the THF-II riboswitch, we determined crystal structures of the THF-II riboswitch from *Mesorhizobium loti* in a ligand-bound state. The RNA in the ligand–RNA complexes adopts a long rod-like, continuously stacked helix structure stabilized by two helices of P1 and P2 and the interconnecting ligand-bound binding pocket. The pterin moiety of the ligands docks into the binding pocket by forming hydrogen bonds with two highly conserved junctional pyrimidines of C22 and U44 in J12 and J21 that connect P1 and P2, respectively, which resembles the hydrogen-bonding pattern at the site FA_PK_ in THF-I riboswitch. Interestingly, the RBS sequence on the right arm of helix P1 forms three non-canonical base pairs with the anti-RBS sequence on the left arm of helix P1, indicating that the ligand-bound riboswitch is in a genetic ‘OFF’ state. Using small-angle X-ray scattering (SAXS) and isothermal titration calorimetry (ITC), we characterized the THF-II riboswitch in solution and reveal that THF binding to the riboswitch is Mg^2+^ dependent, and Mg^2+^ is essential for pre-organization of the binding pocket for efficient ligand binding. DNA oligonucleotide-directed RNase H cleavage assay indicates that ligand binding reduces the accessibility of the RBS on the right arm of P1, thus down-regulating the expression of downstream genes. Together, these results provide mechanistic insights into translation initiation regulation by the THF-II riboswitch.

## MATERIALS AND METHODS

### Ligands

THF, 6-biopterin, sapropterin dihydrochloride (H4B), 7,8-dihydroneopterin (NPR) and methyltetrahydrofolic acid (5-methyl-THF), were all purchased from MedChemExpress (NJ, USA). 7-Deazaguanine (7DG) and folinic acid were purchased from Macklin Biochemical Co., Ltd (Shanghai, China).

### RNA sample preparation

The plasmid encoding an upstream T7 promoter and the RNA sequence of the THF-II riboswitch from *M. loti* was total gene synthesized and sequenced by Wuxi Qinglan Biotechnology Inc., Wuxi, China. Using this plasmid as a template, plasmids encoding the respective RNA mutants were further constructed and confirmed by DNA sequencing.

All the RNAs were prepared by *in vitro* transcription using homemade T7 RNA polymerase. The double-stranded DNA fragment templates for *in vitro* RNA production were generated by polymerase chain reaction (PCR) using an upstream forward primer targeting the plasmids and a downstream reverse primer specific to the respective cDNAs. To ensure 3′ homogeneity of the transcription product, two 2′-methoxy modifications were introduced to the 5′ end of the reverse primers (26). *In vitro* transcription was carried out at 37°C for 2–3 h in a water bath. The transcription supernatants were directly applied to a HiLoad 16/600 Superdex 75 gel filtration column and the RNAs were purified by size exclusion chromatography (SEC). The SEC buffer contains 20 mM Tris–HCl pH 7.5, 100 mM KCl, 10 mM MgCl_2_ and 5 mM dithiothreitol (DTT). Fractions containing the target RNAs were pooled and concentrated with Amicon Centrifugal Filter Units. Concentrated RNAs were stored at –80°C until use. The concentrations of RNA were determined by UV-Vis absorbance at 260 nm using a NanoDrop 2000 (Thermo Scientific). The molar extinction coefficients of RNAs were calculated from the primary RNA sequences using the OligoAnalyzer Tool (https://sg.idtdna.com/calc/analyzer).

### Isothermal titration calorimetry

ITC experiments were performed at 25°C on a MicroCal PEAQ-ITC microcalorimeter at the High Throughput Screening (HTS) Core Facility, Center of Pharmaceutical Technology, Tsinghua University. To test the effects of MgCl_2_ on binding activity between RNAs and ligands, the THF-II riboswitch wild-type (WT) and mutant RNAs were buffer-exchanged into buffers containing 20 mM Tris–HCl pH 7.5, 100 mM KCl, 1 mM Tris (2-carboxyethyl) phosphine (TCEP) supplemented with different concentrations of MgCl_2_ (0–10 mM) using SEC. About 280 μl and 30 μM RNA samples in each buffer were loaded into the sample cell. The syringe cell was filled with ∼45 μl of 0.75 mM ligand dissolved in the same buffer. The ligands were then titrated into the RNA solution with an initial 0.4 μl injection, followed by 19 serial 2 μl injections, with 120 s spacing time between each injection. The reference power was set as 10 μcal/s. The background data obtained from the buffer sample were subtracted before the data analysis. Integrated heat data were analyzed using the Origin7 software package provided by the manufacturer using a ‘one set of sites’ binding model. All the binding constants and thermodynamic parameters are listed in Supplementary Table S1.

### Crystallization

Crystals were obtained for the THF-II-loti_TL_ RNA construct in the presence of its various ligands (THF, H4B, NPR and 7DG) and its C22G mutant. For the THF-II-loti_TL_ construct, a final RNA concentration of 150 μM was mixed with THF, H4B, NPR or 7DG in a molar ratio of 1:5 and kept on ice for ∼1 h, followed by centrifugation at 13 000 rpm for 10 min at 4°C prior to crystallization. For its C22G mutant, the RNA at a concentration of 150 μM was directly used for crystallization.

Crystallization experiments were performed by mixing 2 μl of RNA–ligand complex or the C22G mutant RNA with 2 μl of reservoir solution using the hanging-drop vapor diffusion method at 16°C. For the THF-II-loti_TL_–ligand complex, the best crystals appeared in 0.1 M sodium citrate pH 6.5, 1.5–2.5 M ammonium sulfate within 5 days, and would grow up to 300 × 100 × 100 μm^3^ in 1–2 weeks. For its C22G mutant, the best crystals appeared in 20 mM HEPES pH 7.5, 20–25% polyethylene glycol (PEG) 3350, and would grow up to 200 × 200 × 50 μm^3^ within 1 week.

### Structure determination and refinement

All X-ray diffraction data were collected on beamline BL18U1 or BL19U1 at the Shanghai Synchrotron Radiation Facility (SSRF) and processed with XDS (27,28) or HKL2000 (HKL Research). To solve the phase problem, selenourea (Se-urea) was used as a source of anomalous signal (29). Crystals of the THF-II-loti_TL_–THF complex were fished out and soaked in the crystallization buffer supplied with 0.2 M Se-urea and 20% glycerol for 1 min and flash-frozen in liquid nitrogen, then kept in liquid nitrogen until diffraction. To avoid oxidation of Se-urea, 1 M sodium sulfite was used to dissolve Se-urea to the final concentration of 2 M. The AutoSol program in the Phenix suite was used for the single-wavelength anomalous diffraction (SAD) method (30). Finally, four selenium atoms were located. The model was further built in COOT (31) and refined using the phenix.refine program in the Phenix suite (30). The structures of THF-II-loti_TL_ in complex with other ligands (H4B, NPR or 7DG) and the C22G mutant were solved by molecular replacement (MR) using the Phaser-MR program in the Phenix suite with the structure of THF-II-loti_TL_ as the initial model. All X-ray data collection and crystallographic refinement statistics are listed in Supplementary Table S2.

### Small-angle X-ray scattering

All the parameters for data collection and software employed for data analysis are similar to those described before (32). All the RNA samples were purified by SEC in a buffer containing 20 mM Tris–HCl pH 7.5, 100 mM KCl, various concentrations of MgCl_2_, 3% glycerol and 5 mM DTT. SAXS measurements were carried out at room temperature at the beamline 12 ID-B of the Advanced Photon Source, Argonne National Laboratory. The scattered X-ray photons were recorded with a PILATUS 2M detector (Dectris). The set ups were adjusted to achieve scattering *q*-values of 0.005 < *q* < 0.8/Å, where *q* = (4π/λ)sin(θ), and 2θ is the scattering angle. Thirty two-dimensional (2D) images were recorded for each sample solution or corresponding buffer using a flow cell, with an exposure time of 1 s. No radiation damage was observed as confirmed by the absence of systematic signal changes in sequentially collected X-ray scattering images. The 2D images were reduced to 1D scattering profiles using MatlabR2017a. Scattering profiles of the RNAs were calculated by subtracting the background buffer contribution from the sample buffer profile using the program PRIMUS3.2 (33) following standard procedures. The forward scattering intensity *I*(0) and the radius of gyration (*R*_g_) were calculated at low *q* values in the range of *qR*_g_ <1.3, using the Guinier approximation: ln*I*(*q*) ≈ ln(*I*(0)) – *R*_g_^2^*q*^2^/3. These parameters were also estimated from the scattering profiles with a broader *q* range of 0.006–0.30/Å using the indirect Fourier transform method implemented in the program GNOM4.6 (34), along with the PDDF (pair distance distribution function) and the maximum dimension of the molecule, *D*_max_. The parameter *D*_max_ (the upper end of distance *r*) was chosen so that the resulting PDDF has a short, near-zero value tail to avoid underestimation of the molecular dimension and consequent distortion in low-resolution structural reconstruction. The volume of correlation (*V*_c_) was calculated using the program Scatter, and the molecular weights of solutes were calculated on a relative scale using the *R*_g_/*V*_c_ power law developed by Rambo *et al.* (35), independently of RNA concentration and with minimal user bias. Low-resolution bead models were built up with the program DAMMIN, which generates models represented by an ensemble of densely packed beads (36), using scattering data within the *q* range of 0.006–0.30/Å.

### Oligonucleotide-directed RNase H cleavage assay

The assay was performed by following the procedures in previous work (37). All experiments were done with SEC-purified RNA samples generated by *in vitro* transcription in a buffer containing 20 mM Tris–HCl pH 7.5, 100 mM KCl, 10 mM MgCl_2_ and 1 mM TCEP. Four RNA samples in a final concentration of 10 μM (20 μl) were incubated with 1 mM THF or an equal volume of dimethylsulfoxide (DMSO) for 30 min on ice. After incubation, 2 μl of 10× RNase H reaction buffer, 2 μl of DNA oligonucleotide (5′-CGTCTCCCGT-3′, 100 μM) or RNA storage buffer, 0.2 μl of RNase H (NEB) or 0.2 μl of 10× reaction buffer was added to samples and digested at 37°C for 30 min. Digestion was terminated by addition of 1 μl of 0.5 M EDTA. A 10% denaturing polyacrylamide gel was used to resolve RNAs and then stained by GelSafe nucleic acid stain. The percentage of cleavage product relative to the total amount of cleaved and full-length RNA was calculated using Image J 1.53a. All experiments were carried out in at least triplicate.

## RESULTS

### Construct design and crystallization of the THF-II riboswitch

**Figure 1.**
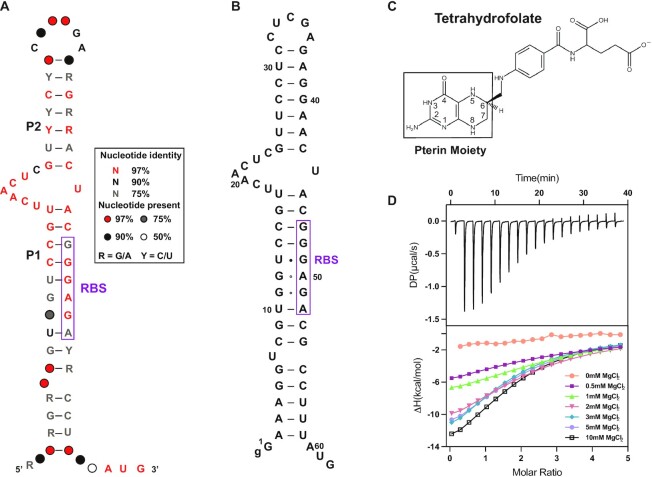
Predicted secondary structure model for the *folE* motif RNAs and ITC experiments for THF binding to THF-II-loti_62_ in various Mg^2+^ concentrations. (**A**) Consensus sequence and secondary structure model for the *folE* motif RNAs. The predicted RBS is highlighted in a purple box, and the predicted AUG translation start site is highlighted in red. (**B**) Sequence and predicted secondary structure model of THF-II-loti_62_ from *M. loti*. The predicted RBS is highlighted in a purple box. (**C**) Chemical structure of THF. The pterin moiety is highlighted with a black box. (**D**) Thermogram of the ITC experiment of THF-II-loti_62_ RNA binding to THF (top) and overlay of integrated fitted heat plots in various Mg^2+^ concentrations (bottom); for the arithmetic mean of the *K*_D_ value and thermodynamic parameters, see Supplementary Table S1.

Recently, the consensus sequence and a secondary structure model for a total of 86 unique *folE* motif RNAs have been reported (24), revealing the presence of most of the conserved nucleotides within an asymmetric internal bulge joining the two base-paired substructures, called P1 and P2 (Figure 1A). The 62 nt *folE* motif RNA from *M. loti* has been validated biochemically as a THF-II riboswitch (dubbed here as THF-II-loti_62_) (Figure 1B) (24). Mg^2+^ ions are known to be important for the folding and function of many RNAs including riboswitches (38–40). To better understand how Mg^2+^ affects the binding of ligand THF (Figure 1C) to the THF-II riboswitch, ITC measurements were first performed over Mg^2+^ concentrations from 0 to 10 mM (Figure 1D). Interestingly, the binding of THF to THF-II-loti_62_ is highly dependent on Mg^2+^. While no THF binding can be observed in the absence of Mg^2+^, THF binds to THF-II-loti_62_ with moderate affinities at physiological Mg^2+^ concentrations, and the binding affinities become higher as the Mg^2+^ concentration increases (Supplementary Table S1). The binding of THF-II-loti_62_ to THF in 10 mM MgCl_2_ was measured with a 1:1 stoichiometry and an apparent dissociation constant of ∼26 μM by ITC (Supplementary Table S1), which is consistent with the previous in-line probing data (24). Thus, Mg^2+^ is essential to proper folding of the THF-II riboswitch for efficient ligand binding.

**Figure 2.**
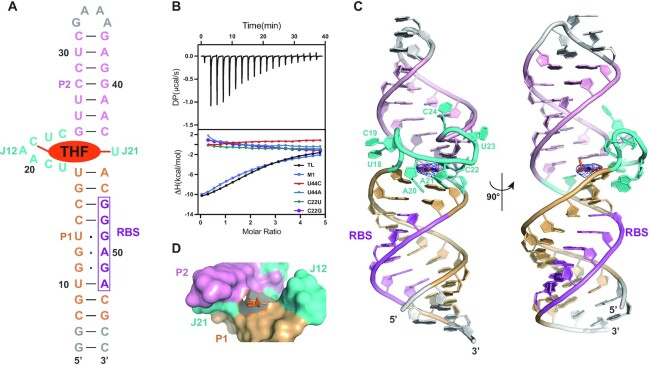
Secondary and tertiary structure of the THF-II-loti_TL_ RNA bound to THF. (**A**) The secondary structure of THF-II-loti_TL_ is derived from the crystal structure. Residues are numbered according to THF-II-loti_62_ WT RNA. Helix P1 is colored in gold, junctions J12 and J21 are colored in cyan and helix P2 is colored in light pink. Residues different from that in THF-II-loti_62_ RNA are colored in gray. THF is highlighted in an orange ellipse. (**B**) Thermogram of a representative ITC experiment of THF-II-loti_TL_ RNA binding to THF (top) and overlay of integrated fitted heat plots of THF-II-loti_TL_ RNA and its M1, U44C, U44A, C22U and C22G mutants (bottom); for the arithmetic mean of *the K_D_* value and thermodynamic parameters, see Supplementary Table S1. (**C**) Crystal structure of THF-II-loti_TL_ bound with THF (shown in sticks) in cartoon representation in two different views. The RBS sequence is highlighted in purple. The composite omit maps (contoured at the 1.0 σ level) of the ligand are shown in tv_blue mesh. (**D**) Surface representation of the binding pocket. THF (shown in sticks) is labeled in orange. The color codes in (C) and (D) are the same as that in (A).

We used the THF-II-loti_62_ construct for initial crystallization screening but this yielded no crystal hit. Based on the consensus sequence and secondary structure model of the THF-II riboswitch (Figure 1A), a subconstruct of THF-II-loti_62_, in which the apical loop of P2 was replaced with a GAAA tetraloop, and the single-nucleotide bulge U7 and the lower end of P1 containing three A–U base pairs were deleted, was designed and named THF-II-loti_TL_ to facilitate crystallization (Figure 2A). The binding affinity of THF-II-loti_TL_ for THF in 10 mM MgCl_2_ was also determined by ITC (Figure 2B), which is comparable with that of THF-II-loti_62_ (Supplementary Table S1), indicating that such optimization did not affect the ligand binding. Though no crystal was obtained for THF-II-loti_TL_ RNA alone, we obtained crystals for THF-II-loti_TL_ in complex with THF that diffracted to 2.85 Å. The space group was *P*3_1_21, in which each asymmetry unit contained only one molecule. We solved the structure with the SAD phasing method by collecting the anomalous signal from Se-urea-soaked crystals (Supplementary Figure S1). Previously, Se-urea has been utilized to provide an anomalous signal for the SAD phasing of proteins and DNA crystals (29), but its applicability for RNA crystals has not been tested. By collecting X-ray diffracting data of Se-urea-soaked RNA crystals, we located four Se-urea molecules, which provide strong anomalous signals enough for initial model building (Supplementary Figure S1). All these four Se-urea molecules interact with RNA by forming hydrogen bonds via the selenium atoms. Their occupancies are 41, 34, 32 and 40%, respectively. The root mean square differentiation (RMSD) between the Se-urea-soaked crystal structure and the native structure is 0.4 Å. The X-ray crystallographic statistics are provided in Supplementary Table S2.

### Overall structure of the THF-II riboswitch in complex with THF

The overall 3D structure of THF-II-loti_TL_ RNA in complex with THF has a long rod-like shape (Figure 2C), in which the two helices P1 and P2 exhibit coaxial stacking mediated by the interconnecting junctions J12 and J21. The proximal base pairs at the interface between P1 and P2 along with the junctions J12 and J21 form a semi-open ligand-binding pocket at the center of the RNA (Figure 2C). The pterin moiety of the ligand THF inserts into the binding pocket and no interactions are observed between the remaining part of THF and RNA (Figure 2D). Notably, three non-canonical base pairs (U13·G49, G12·A50 and G11·G51) zipper up the RBS sequence on the right arm of helix P1 (Supplementary Figure S2), indicating that the riboswitch is in a genetic ‘OFF’ state in the presence of ligand, and thus down-regulating expression of downstream genes.

### Structure of the ligand-bound binding pocket

**Figure 3.**
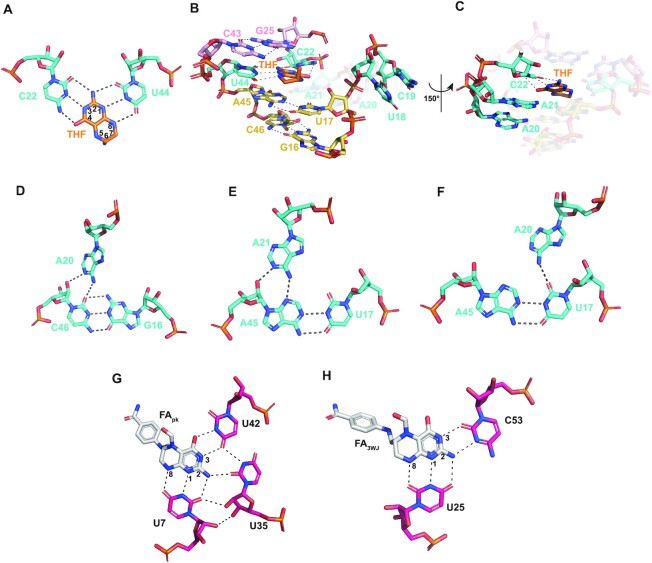
Structural details of ligand recognition by the THF-II riboswitch. (**A**) Details of ligand–RNA interactions between THF-II-loti_TL_ and THF. C22 and U44 form three hydrogen bonds with the N2/N3 edge and N1/N8 edge of the pterin moiety of THF, respectively. (**B** and **C**) Structural details of the ligand-binding pocket. The C22–THF–U44 base triplet is sandwiched between base pair G25–C43 and two base triplets of G16–C46·A20 and U17–A45·A21 (B). The first two nucleotides of J12, U18 and C19, are stacked together (B). Continuous stacking of A20, A21 and C22 fixes the orientation of C22 for pterin recognition (C). (**D**) A-minor interaction between A20 and the C46–G16 base pair. N6 (amino) and N1 of A20 form one hydrogen bond with O2 (carbonyl) and O2' (hydroxyl) of C46, respectively. (**E**) A-minor interaction between A21 and A45–U17 base pair. N6 (amino) and N1 of A21 each form one hydrogen bond with N3 and O2' (hydroxyl) of A45. (**F**) A-minor interaction between A20 and A45–U17 base pair. N6 (amino) of A20 forms one hydrogen bond with O2 (carbonyl) of U17. (**G**and **H**) Details of interactions between the THF-I riboswitch and ligands at sites of the pseudoknot (FA_pk_) (H) and three-way junction (FA_3WJ_) (I). PDB code: 3SD1.

When the ligand THF is bound at the center of the RNA, its pterin ring is fixed in place by formation of hydrogen bonds with C22 in J12 and U44 in J21 (Figure 3A) and sandwiched below a long-range base pair (G25·C43) and above two base triplets of G16–C46·A20 and U17–A45·A21 that are at the proximal ends of the P1 and P2 helices, respectively (Figure 3B). As shown in Figure 1A, all the residues involved in the formation of ligand-binding pockets are highly conserved, consistent with their functional importance. The importance of the integrity of ligand-binding pockets was further supported by ITC experiments on four RNA mutants, in which C22U, C22G, U44C or U44A mutations all cause the loss of binding to the THF ligand (Figure 2B). The ligand-bound binding pocket of the THF-II riboswitch is stabilized by a series of tertiary interactions. First, the bases of U18 and C19 are flipped out of the binding pocket but stacked together, causing a 180° turn of the J12 strand direction (Figures 3B and 2C). Second, the bases of A20, A21 and C22 are also stacked together (Figure 3C), which may stabilize the orientation of C22 for proper base-pairing interaction with the pterin moiety of THF. Both A20 and A21 exhibit A-minor interactions, which use their Watson–Crick edges to pair with the sugar edges of C46 and A45, respectively (Figure 3D–F). The C46 and A45, on the other hand, also form canonical Watson–Crick base pairs with G16 and U17, respectively, thus forming two base triplets of G16–C46·A20 and U17–A45·A21 to stack with the C22–THF–U44 base triplet. Third, though the bases of U23 and C24 are randomly flipped out and no obvious stacking between them is observed, they cause another 180° turn of the strand direction, which turns the junction back to the orientation of the helix (Figure 2C). Last, but not least, the canonical Watson–Crick base pair G25·C43 at the proximal end of P2 stacks on U44 in J21, and may stabilize the base orientation of U44 and reinforce interaction between U44 and the pterin moiety of THF (Figure 3B). Altogether, the continuous base pair and base triplet stacking across the P1, P2 and ligand-bound binding pocket facilitate the formation of a long rod-like helix globally.

### Molecular basis for ligand recognition by the THF-II riboswitch

In the crystal structure of the holo THF-II riboswitch, electron density can be clearly defined for the pterin moiety but not for the benzoate ring and the glutamyl moiety of the THF ligand (Figure 2C, D); in contrast, densities for both the pterin and benzoate ring except for the glutamate moieties can be observed in structures of the holo THF-I riboswitch (21,22). This observation suggests the important role of the pterin moiety in RNA binding for both types of riboswitches. The surface representation of the THF-II riboswitch ligand-bound pocket clearly shows that the pterin moiety of THF (depicted in sticks) intercalates between P1 and P2 and becomes almost completely buried with the help of the nucleotides from junction J12 (Figure 2D).

A detailed inspection of the hydrogen-bonding interaction modes between the THF pterin moiety and RNAs reveals similarities and differences between the THF-I and THF-II riboswitches (Figure 3A, G, H). There are two ligand-binding sites (FA_PK_ and FA_3WJ_) identified in the structure of the THF-I riboswitch, in which the ligand is bound to the minor groove face of each binding site. While the N1/N8 edge of the pterin moiety forms three hydrogen-bonding interactions with U7 in site FA_PK_ and U25 in site FA_3WJ_, respectively, the N2/N3 edge of the pterin moiety forms three hydrogen bonds with two uridines (U35 and U42) in site FA_PK_ but two hydrogen bonds with C53 in site FA_3WJ_, respectively (Figure 3G, H). In the structure of the THF-II riboswitch, only one ligand is bound to the major groove face of the site formed between C22 in J12 and U44 in J21 (Figure 3A). The N1/N8 edge of the pterin moiety forms three hydrogen-bonding interactions with U44, whereas the N2/N3 edge of the pterin moiety forms three hydrogen bonds with C22 (Figure 3A), in contrast to the formation of only two hydrogen bonds in recognition of C53 at site FA_3WJ_ in the THF-I riboswitch. At all three THF-binding sites, though the pterin moiety uses the same N1/N8 edge to recognize highly conserved uridine, the donors and acceptors of hydrogen bonds are not uniform. For example, the donation of a hydrogen bond is from N8 of the ligand to O2 of U25 in site FA_3WJ_ but from N2 of the ligand to O2 of U7 in site FA_PK_ of the THF-1 riboswitch across the N1/N8 edge of the ligand, whereas it is from N2 of the ligand to O2 of U35 at site FA_PK_ but from N2 of the ligand to N3 of C53 at site FA_3WJ_ across the N2/N3 edge of the ligand (Figure 3G, H). The hydrogen-bonding pattern for THF–RNA interaction in the THF-II riboswitch is more similar to that at the site FA_PK_ of the THF-I riboswitch. The donation of a hydrogen bond is from N2 of the ligand to O2 of U44 across the N1/N8 edge and from N2 of the ligand to O2 of C22 across the N2/N3 edge in the THF-II riboswitch (Figure 3A). Interestingly, a previous report suggested that RNA–ligand interactions at site FA_PK_ in the THF-I riboswitch are more functionally relevant (23).

**Figure 4.**
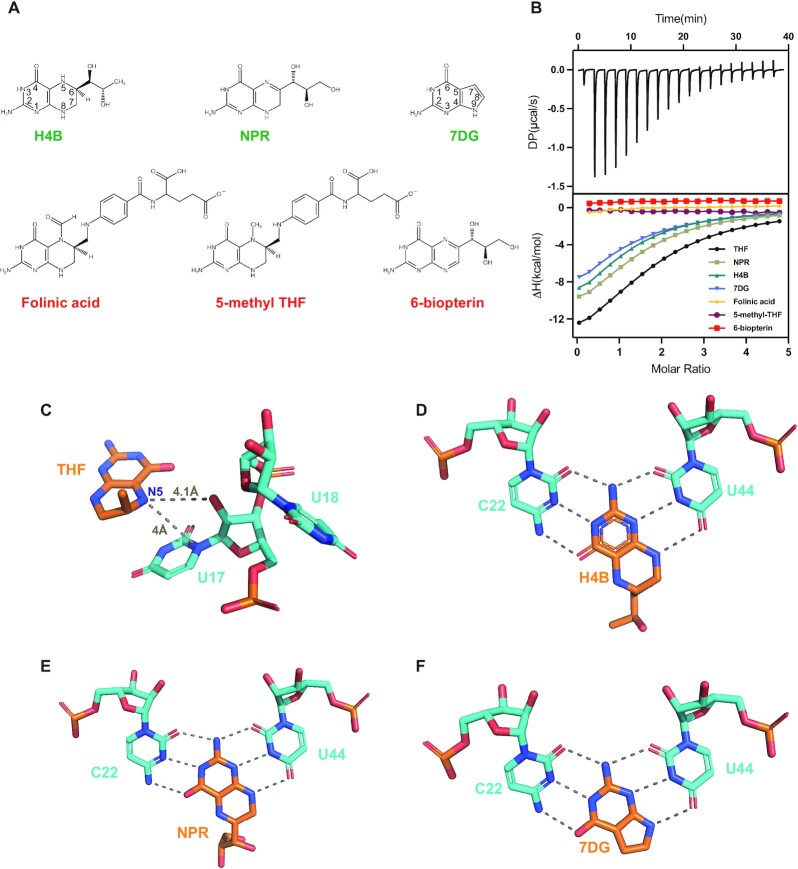
Interactions between the THF-II riboswitch and THF analogs. (**A**) Chemical structures of the THF analogs involved in this study. Molecule names are colored in green if they are bound by the RNA; otherwise they are in red. (**B**) Thermogram of a representative ITC experiment of THF-II-loti_62_ binding to THF (top) and overlay of integrated fitted heat plots of THF-II-loti_62_ binding to THF, NPR, H4B, 7DG, folinic acid, 5-methyl-THF and 6-biopterin (bottom); for the arithmetic mean of the*K*_D_ value and the thermodynamic parameters, see Supplementary Table S1. (**C**) Potential steric block between the N5 position of the pterin moiety (shown in sticks, orange) of the ligand and U17 of the riboswitch, whose distances are within 5 Å. (**D–F**) Structural details of ligand–RNA interactions between THF-II-loti_TL_ and H4B (D), NPR (E) and 7DG (F).

To assess the ligand specificity of the THF-II riboswitch, we measured its binding affinities for six analogs of THF or purine by ITC (Figure 4A). Among these analogs, H4B and NPR exhibit a similar binding affinity to THF, whose structural differences only lie in C6 of the pterin moiety in H4B and an oxidized N5 in NPR, respectively (Figure 4B). 7DG binds to the RNA but with a relatively weaker binding affinity than THF, whereas the RNA fails to bind 5-methyl-THF, folinic acid and 6-biopterin. These results are consistent with a previous in-line probing analysis (24). In the structure of the THF-II-loti_TL_–THF complex, the N5 position of the pterin is within 5 Å to the O2′ and C2′ atoms of U17 (Figure 4C), suggesting that the aptamer uses a steric block to discriminate against 5-methyl-THF and folinic acid which carry chemical moieties larger than a proton at the N5 position. The N8 of 6-biopterin is in an oxidized form, so it loses the ability to form a hydrogen bond with O4 of U44 (Figure 4A).

To better understand RNA–ligand interactions, we determined the crystal structures of the THF-II riboswitch in complex with H4B, NPR and 7DG (Supplementary Figure S3A–C). The overall structures of all the complexes are highly similar and their RMSDs from the structure of the THF–RNA complex are <0.5 Å. For H4B and NPR, only the pterin moiety of the ligands can be seen, consistent with the observation that the pterin moiety is mostly responsible for ligand–RNA interactions (Supplementary Figure S3A, B). For 7DG, the hydrogen-bonding pattern with RNA is similar to that of other analogs except for the planar structure of the second ring (Supplementary Figure S3C). At the binding pocket, all ligands form six hydrogen bonds with the RNA (Figure 4D–F). As the N5 position of the NPR pterin is not involved in RNA binding, though in an oxidized form, it will not affect NPR binding to RNA (Figure 4A, E). The N9 position of the second five-carbon ring of 7DG also forms a hydrogen bond with O4 of U44 of the RNA (Figure 4F).

### Crystal structure of the C22G mutant of the THF-II riboswitch

**Figure 5.**
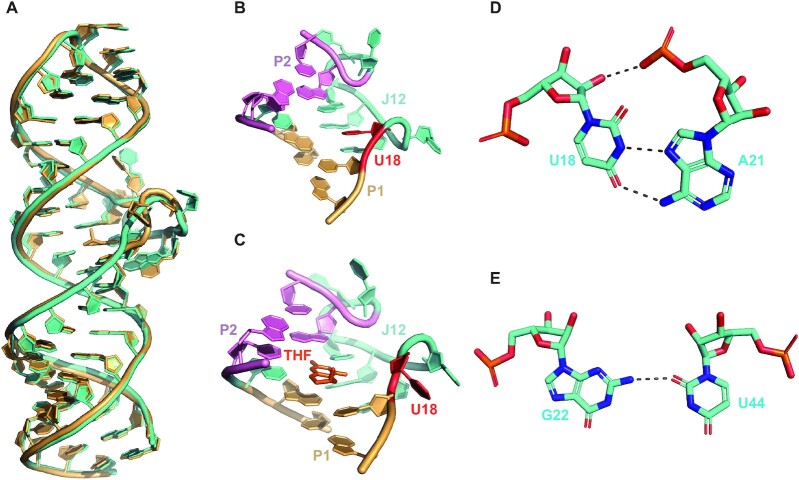
Crystal structure and ligand-binding pocket architecture of the THF-II-loti_TL_-C22G mutant. (**A**) Structure overlay of the THF-II-loti_TL_–THF complex (shown in gold) and the THF-II-loti_TL_-C22G mutant (shown in aquamarine). The RMSD is 0.7 Å. (**B**and **C**) Close-up views of the binding pocket architecture of the THF-II-loti_TL_-C22G mutant (B) and the THF-II-loti_TL_–THF complex (C). Helix P1 is colored in gold, junction J12 is colored in cyan, helix P2 is colored in pink, U18 is highlighted in red and THF is highlighted in orange. (**D**) Hoogsteen base pairing between U18 and A21 in THF-II-loti_TL_-C22G mutant. O2' (hydroxyl) of U18 and OP2 of U21, N3 (imino) of U18 and N7 of A21, and O4 (carbonyl) of U18 and N6 (amino) of A21 form one hydrogen bond, respectively. (**E**) Interaction between G22 and U44. The C2 (amino) of G22 forms one hydrogen bond with O2 (carbonyl) of U44.

A previous in-line probing analysis suggested that the C22G mutant of the THF-II riboswitch alone exhibits a similar structure to the WT RNA in complex with ligand (24). Our ITC experiments indicated that the C22G mutant lost the binding activity to THF (Figure 2B). We then determined the crystal structure of the C22G mutant of THF-II-loti_TL_ RNA (THF-II-loti_TL_-C22G), which diffracted to 3.22 Å. The space group of the crystal is *P*3_1_21 (Supplementary Table S2), in which one asymmetry unit contains only one RNA molecule. The overall crystal structure of THF-II-loti_TL_-C22G is highly similar to that of WT RNA, between which the RMSD is 0.7 Å (Figure 5A). Their P1 and P2 helices are almost identical. The structural differences mainly lie in the J12 junction loop (Figure 5B, C). In the C22G mutant, U18 intercalates into the binding pocket and forms a Hoogsteen base pair with A21 (Figure 5B, D). In contrast, U18 in WT RNA is flipped out of the binding pocket and stacked with C19 (Figure 5C). Furthermore, the N2 of G22 forms one hydrogen bond with O2 (carbonyl) of U44 (Figure 5E), mimicking the ligand-bound conformation in WT RNA. Interestingly, a hydrogen bond is formed between O2' (hydroxyl) of U18 and OP2 of A21 (Figure 5D), which may position the O2' (hydroxyl) of U18 in an in-line position, thus explaining a previous observation from in-line probing that the C to G mutation results in a similarly robust cleavage product at U18 to WT RNA (24).

### Mg^2+^- and ligand-induced conformational changes of the THF-II riboswitch

**Figure 6.**
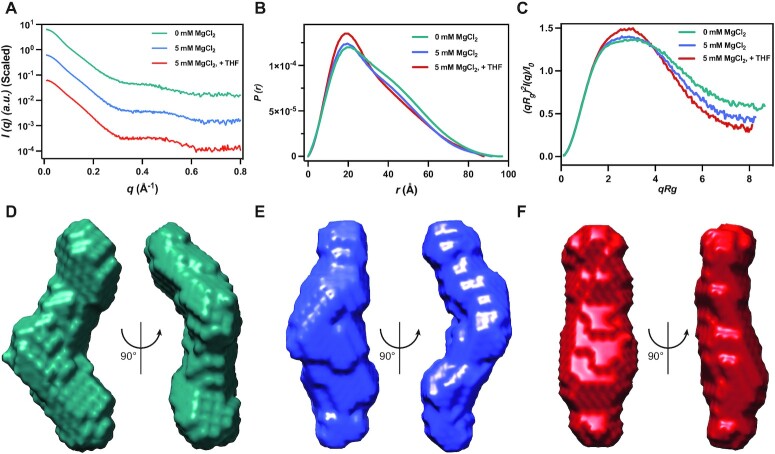
SAXS analysis of THF-II-loti_62_ in response to Mg^2+^ and ligand binding. (**A–C**) The scattering curves (A), PDDFs (B) and dimensionless Kratky plots (C) of THF-II-loti_62_ at various conditions. (**D–F**) The *ab initio* shape envelopes of THF-II-loti_62_ in two views in 0 mM MgCl_2_ and ligand-free conditions (D), in 5 mM MgCl_2_ and ligand-free conditions (E) and in 5 mM MgCl_2_ and ligand-bound conditions (F).

As crystallization trials for aligand-free THF-II riboswitch were not successful and Mg^2+^ is essential for efficient ligand binding of the THF-II riboswitch (Figure 1D), we characterized the THF-II-loti_62_ RNA in the absence and presence of Mg^2+^ and THF ligand in solution using SAXS. The scattering profiles, with scattering intensity *I*(*q*) plotted against momentum transfer *q*, the pair distance distribution function PDDFs and the dimensionless Kratky plots transformed from the scattering profiles for the THF-II-loti_62_ RNA are shown in Figure 6A–C. The structural parameters of the radius of gyration (*R*_g_) and maximum end-to-end distance (*D*_max_) become smaller upon the increase of Mg^2+^ concentration (Supplementary Table S3), indicating that the riboswitch RNA becomes more compact upon Mg^2+^ binding. In the presence of 5 mM Mg^2+^, the binding of THF results in further compaction of the riboswitch. This observation is consistent with the changes in the dimensionless Kratky plots (Figure 6C). The dimensionless Kratky plots of THF-II-loti_62_ under different conditions exhibit a common single maximum, but the normalized (*q ×R*_g_)^2^*I_q_*/*I*_0_ values at higher *q*× *R*_g_ become smaller upon Mg^2+^ and THF binding, suggesting improved folding and reduced flexibility of the riboswitch upon Mg^2+^ and THF binding (Figure 6C).

To gain more specific information on the structure of THF-II-loti_62_ under different conditions, *ab initio* shape envelopes were built using the program DAMMIN (Figure 6D–F). In the absence of Mg^2+^, under which condition the THF-II riboswitch loses its ability to bind THF, the THF-II riboswitch exhibits a highly bent envelope (Figure 6D). In contrast, the envelope of the riboswitch becomes extended in the presence of 5 mM Mg^2+^ (Figure 6E), indicating that the coaxial stacking architecture of THF-II riboswitch RNA is partially formed. Upon further addition of THF in the presence of Mg^2+^, the riboswitch exhibits an extended rod-like shape and is more rigid, suggesting the formation of similar coaxial stacking to that observed in the ligand-bound crystal structure (Figure 6F). These results indicate that the formation of the coaxially stacked architecture of the THF-II riboswitch highly depends on simultaneous Mg^2+^ ions and ligand binding.

### Ligand binding reduces RBS accessibility of the THF-II riboswitch

**Figure 7.**
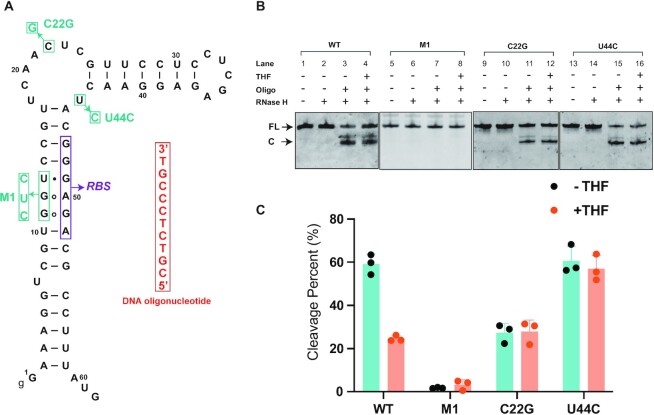
DNA oligonucleotide-directed RNase H cleavage of THF-II-loti_62_. (**A**) THF-II-loti_62_ sequence used for DNA oligonucleotide-directed RNase H cleavage assay. Mutation sites are boxed and labeled in cyan. To be more specific, three non-canonical base pairings on the RBS were all changed to Watson–Crick base pairs on the helix P1 for the M1 mutant. The RBS sequence is boxed in purple and the DNA oligonucleotide is boxed in red on the right. (**B**) Denaturing PAGE gel analysis of RNase H cleavage products of the WT, M1, C22G and U44C. This figure was assembled from several gels. FL, full-length transcripts; C, RNase H cleavage products; WT, wild-type THF-II-loti_62_ RNA. (**C**) Quantification of RNase H cleavage products. The values are plotted to represent the means ± standard deviations of at least three independent experiments for each construct.

To establish the effect of ligand binding on the accessibility of the putative RBS, we performed RNase H cleavage assay using a DNA probe that targeted the right arm of P1 containing the RBS of THF-II-loti_62_ (Figure 7A) (37,41). As a control, RNA alone was not cleaved by RNase H (Figure 7B, lanes 2, 6, 10 and 14). In the absence of THF but presence of the DNA oligo, ∼60% of the WT THF-II riboswitch RNA was efficiently cleaved by RNase H (Figure 7B, lane 3 and Figure 7C, column 1), indicating that the RBS region was highly accessible for the DNA probe; in the presence of both the DNA probe and ligand THF, only ∼25% of the RNA was cleaved by RNase H (Figure 7B, lane 4 and Figure 7C, column 2). These observations suggest that the THF-II riboswitch reduces the accessibility of the RBS region to the DNA probe in response to ligand binding.

Three mutant constructs were designed to investigate the structural basis of RBS accessibility in response to THF binding. In the M1 mutant, three nucleotides of the anti-RBS sequence on the left arm of P1 were mutated to the complementary sequence of the RBS (GGU→CUC) (Figure 7A), which allows the formation of canonical Watson–Crick base pairs and thus make the RBS helix much more thermodynamically stable. The *K*_d_ value between THF and RNA with the M1 mutation is slightly lower than that of WT RNA (Figure 2B; Supplementary Table S1). In the absence or presence of THF ligand, the M1 mutant RNA is hard to cleave (Figure 7B, lane 7, 8 and Figure 7C, column 3, 4), suggesting that the stabilization of the RBS helix causes inaccessibility of the RBS to the DNA probe. For the C22G mutant in which THF-binding activity is lost, its RNA cleavage percentages decrease to 28% in both the absence and presence of THF ligand (Figure 7B, lane 11, 12 and Figure 7C, column 5, 6), which are comparable with that of the WT RNA in the presence of THF. The ligand unresponsiveness and reduced RBS accessibility are understandable since the C22G mutant loses the ligand-binding activity and can be a mimic of the ligand-bound THF-II riboswitch. For the U44C mutant whose ligand-binding activity is also lost, the RNA cleavage percentages are ∼60% in the absence or presence of THF ligand, which is comparable with that of the WT RNA in the absence of ligand (Figure 7B, lanes 15, 16 and Figure 7C, columns 7, 8). These data suggest that ligand binding and the metastability of the three non-canonical base pairs in helix P1 are crucial for the functional switching of the THF-II riboswitch.

## DISCUSSION

**Figure 8.**
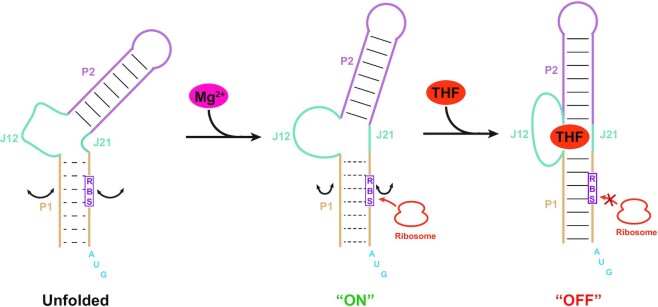
Proposed mechanism of translation initiation regulation by the THF-II riboswitch. In the absence of Mg^2+^ (left), the putative ligand-binding pocket of the THF-II riboswitch is partially or entirely unfolded, resulting in a loss of ligand binding capacity. The riboswitch adopting a flexible conformation and the P1 helix harboring the RBS is less stable, allowing for high accessibility of the RBS for the ribosome. The presence of physiological Mg^2+^ (middle) leads to the pre-organization of the ligand-binding pocket and improves the stability of the P1 helix, resulting in an extended conformation of the RNA and reduced accessibility of the RBS for the ribosome. Efficient ligand binding in the presence of Mg^2+^ (right) promotes continuous coaxial stacking across the RNA and further increases the stability of the P1 helix, resulting in a rod-like RNA conformation and very limited accessibility of the RBS for the ribosome, thus down-regulating gene expression.

A detailed understanding of the regulatory mechanism of the THF-II riboswitch is of great value in the development of new antimicrobial therapeutics. In this work, we determined the crystal structures of the THF-II riboswitch from *M. loti* in complex with a variety of ligands and in its mutated (C22G) form, and studied conformational dynamics of the RNA upon Mg^2+^ and ligand binding and in the mutated forms by ITC, SAXS and DNA oligonucleotide-directed RNase H cleavage assay. Our results demonstrate the importance of tertiary interactions, Mg^2+^ and ligand binding in modulating the structure and dynamics of the THF-II riboswitch for efficient ligand recognition and functional switching, allowing us to propose a mechanistic model for the THF-II riboswitch in the regulation of translation initiation (Figure 8).

The THF-II riboswitch is of particular interest among known riboswitches for its simple architecture and intriguing gene regulation mechanism. Riboswitches are generally composed of an aptamer domain responsible for specific binding to the ligand and an expression domain or expression platform responsible for changing gene expression, which are connected by a switching sequence (17). One example of these riboswitches is the THF-I riboswitch, which consists of an aptamer domain exhibiting an ‘inverted’ three-way junctional architecture and a defined switching sequence connecting the downstream expression platform (21,22). By comparison, the aptamer of the THF-II riboswitch adopts a much simpler helix structure. Though the aptamer domain of the THF-II riboswitch is much smaller than that of the THF-I riboswitch, it encompasses part of its expression platform; in other words, both the aptamer and expression platform of the THF-II riboswitch are merged into a single region (Figure 1A, B). Another riboswitch showing similar characteristic is the SAM-III riboswitch, whose expression platform is fused with the aptamer domain and the RBS sequence directly interacts with ligands to induce switching to a genetic ‘OFF’ state (42,43), indicating that the RBS sequence is an intrinsic part of the ligand-binding aptamer domain. In the THF-II riboswitch, though the RBS sequence on the right arm of helix P1 is adjacent to the binding pocket, no obvious interaction between ligand and RBS sequence was observed, thus it is not an intrinsic part of the aptamer domain. For these two classes of riboswitches, ligand-induced conformational changes in the expression platform are relatively small. For the THF-II riboswitch, binding of THF and its derivatives promotes the formation and enhances the stability of the non-canonical base pairs in helix P1 formed by the 5′-anti-RBS and 3′-RBS, which restricts the accessibility of the RBS sequence, preventing its recognition by the ribosome. In contrast, ligand binding to the aptamer domain generally induces dramatic structural changes in the expression platform of most riboswitches. For instance, the SAM-II riboswitch (44) and preQ1-II riboswitch (45,46) undergo dramatic structural changes in response to ligand binding, such as sequestering the RBS sequence by formation of a PK.

Our results reveal similarities and differences in ligand recognition principles by the aptamers of THF-I and THF-II riboswitches. While up to two ligand-binding sites (sites FA_pk_ and FA_3WJ_) are observed in crystal structures of the ligand-bound THF-I riboswitch (21,22), only the site FA_pk_ appears to be important for gene control, whereas the site FA_3WJ_ is important for folding (23). In contrast, only one ligand-binding site is observed in the crystal structures of the ligand-bound THF-II riboswitch. The recognition of the pterin moiety by the THF-II riboswitch is more analogous to that at the site FA_pk_ in the THF-I riboswitch, which shares similar hydrogen-bonding patterns. The functional relevance of the site FA_pk_ and its ligand recognition pattern in both the THF-I and THF-II riboswitch may not be a coincidence but a common solution for riboswitches. Recently, a common feature of riboswitches observed is that the ligand-binding pocket is almost always situated adjacent to the RNA residues that participate in the most long-range contacts in the RNA structure (47). This is the case for both the THF-I and THF-II riboswitches, in which the PK junction interacts with the switch helix P1 in the THF-I riboswitch and the ligand-bound binding pocket directly stacks on the P1 helix in the THF-II riboswitch.

Our results demonstrate that physiological Mg^2+^ concentrations govern the folding pathway of the THF-II riboswitch towards its native ligand-bound state. The processes of ligand binding to riboswitches can be generally classified into two distinct mechanisms, induced fit and conformational selection, which are commonly referred to as ‘binding first’ and ‘folding first’ processes, respectively (48). In the ‘binding first’ mode, ligand binding to an unfolded conformation promotes folding, whereas in the ‘folding first’ mode, the ligand selects high-affinity, pre-folded structures from an ensemble and shifts the conformational equilibrium toward them. Our ITC and SAXS data showed that the THF-II riboswitch is unable to bind with the ligand and adopts a bent conformation in the absence of Mg^2+^, but the ligand binding affinities become stronger and the global shape of the THF-II riboswitch become elongated as Mg^2+^ increases. These results support that the binding pocket is unfolded in the absence of Mg^2+^ and ligand, and that the pre-organization of the binding pocket induced by Mg^2+^ is a prerequisite for efficient ligand binding and coaxial stacking across helices of P1 and P2 and the interconnecting binding pocket. However, in the presence of Mg^2+^ alone, the helices of P1 and P2 and the interconnecting binding pocket still exhibit some degree of conformational dynamics and the coaxial stacking is not stable, probably due to the lack of the base triplet (C22–THF–U44) formed among the ligand and the junctional pyrimidines as observed in the crystal structures. Ligand binding in the presence of Mg^2+^ further promotes the folding of the binding pocket and stabilizes the continuous stacking across the whole molecule, thus reducing the breathing of helix P1 and preventing ribosome binding to the RBS. Such coupled Mg^2+^-induced folding of an active conformation and ligand binding mechanisms have been observed for several riboswitches including the *btuB* riboswitch from *Escherichia coli* (49–51).

The stability of helix P1 is critical for the switching mechanism of the THF-II riboswitch. Unlike helix P2 formed only by canonical Watson–Crick base pairs, helix P1 consists of two thermodynamically weaker non-canonical base pairs and a G–U wobble base pair, as observed in the ligand-bound crystal structures (Supplementary Figure S2). Our DNA oligonucleotide-directed RNase H cleavage assay showed that ligand binding in the presence of physiological Mg^2+^ reduced RBS accessibility for the WT THF-II riboswitch. It is expected that in the presence of physiological Mg^2+^ only, the P1 helix is breathing thus the RBS is accessible for ribosome binding, allowing translation initiation, but ligand binding in the presence of Mg^2+^ further enhances the stability of helix P1 and promotes continuous stacking across the whole molecule, thus reducing the accessibility of RBS for the ribosome and preventing translation initiation. This is supported by the RBS accessibility data for the U44C mutant and the ligand-bound mimetic C22G mutant, which are comparable with that for ligand-free and ligand-bound THF-II-loti_62_, respectively. As both mutants lose their ligand-binding activities, their RBS accessibilities are not responsive to the presence or absence of THF. Interestingly, the M1 mutant of THF-II-loti_62_ which preserves the ligand binding affinity but the thermodynamic stability of helix P1 is increased by replacing the anti-RBS sequence with the complementary sequences to RBS, exhibits significantly reduced RBS accessibility, but also loses the responsiveness to the native ligand. Thus, the switching efficiency of the THF-II riboswitch depends on the metastability of helix P1. The importance of P1 helix stability for gene regulation has been observed for several riboswitches, including the pbuE adenine riboswitch (52), the *Vibrio vulnificus* adenine riboswitch (53,54) and the THF-I riboswitch (55).

## DATA AVAILABILITY

Atomic coordinates and structure factors for the reported crystal structures have been deposited in the Protein Data Bank under accession codes: 7WI9 (THF-bound, Se-urea-soaked), 7WIB (THF-bound), 7WIF (H4B-bound), 7WII (NPR-bound), 7WIE (7DG-bound), 7WIA (THF-II-C22G).

## Supplementary Material

gkac1257_Supplemental_File
