# Supplementary material for: Structural insights into translation regulation by the THF-II riboswitch
Source: Nucleic Acids Res. 2023 Jan 9;51(2):952–65. doi: 10.1093/nar/gkac1257 (PMC9881143; doi:10.1093/nar/gkac1257)
Supplement: gkac1257_Supplemental_File [file gkac1257_supplemental_file.docx]

**Supplementary Data**

**Structural insights into translation regulation by THF-II riboswitch**

Lilei Xu^1, 2, #^, Yu Xiao^1, #^, Jie Zhang^1, 2^, Xianyang Fang^1, 2, 3*^

^1^Beijing Advanced Innovation Center for Structural Biology, Beijing Frontier Research Center for Biological Structure, School of Life Sciences, Tsinghua University, Beijing 100084, China

^2^Center for Synthetic and Systems Biology, Tsinghua University, Beijing 100084, China

^3^Key Laboratory of RNA Biology, Institute of Biophysics, Chinese Academy of Sciences, Beijing, 100101, China

^*^Correspondence: fangxy@mail.tsinghua.edu.cn, fangxy@ibp.ac.cn

^#^These authors contributed equally.

**Contents**

Supplementary Figures S1-S3

Supplementary Tables S1-S3


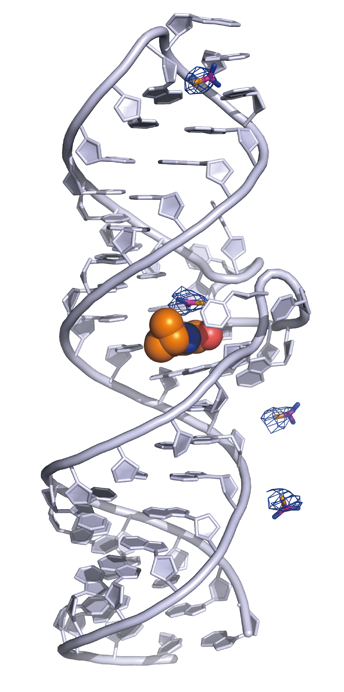


**Figure S1. Difference electron density map for Se-Urea-soaked THF-bounded THF-II-loti_TL_ crystal.** Anomalous electron density map contoured at level 3.0 σ for Se-Urea sites. Se-Urea molecules are labeled in stick representation and selenium atoms, which were used to solve the phase problem of the structure, are colored in orange. THF was in sphere representation.


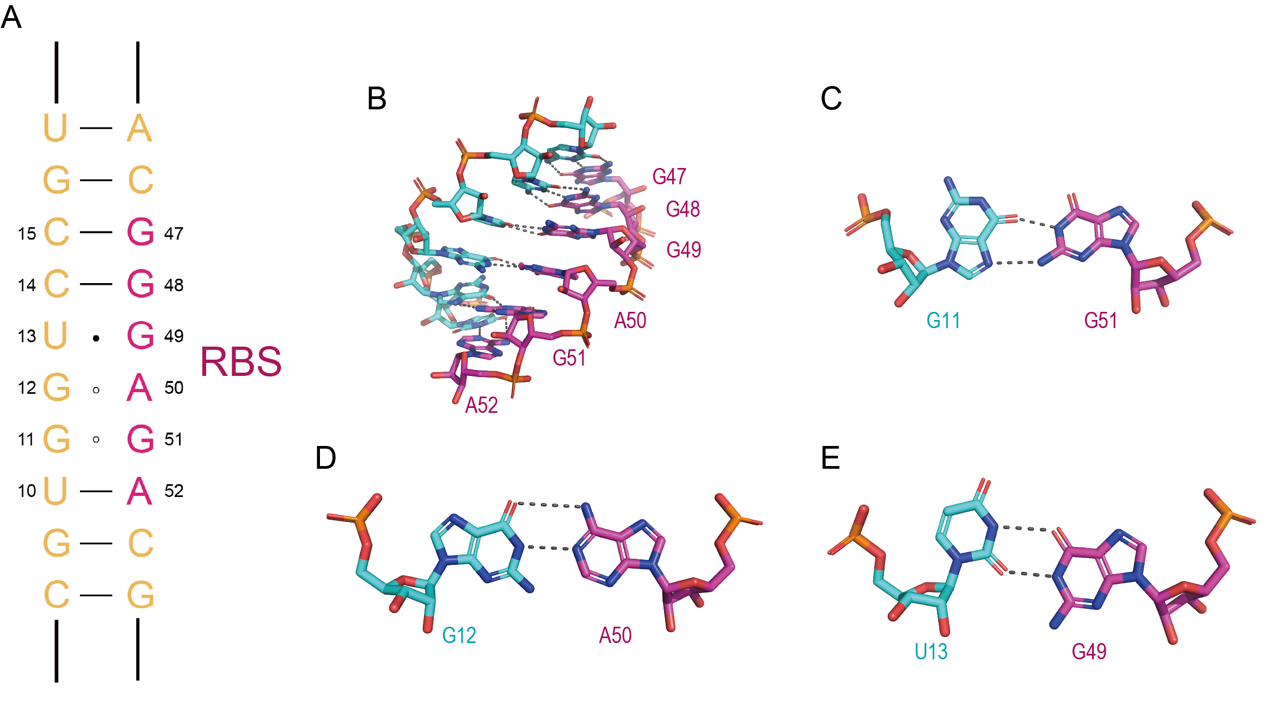


**Figure S2. Formation of RBS helix in the THF-II-loti_TL_-THF complex structure.** (**A**) Schematic secondary structure of the RBS helix of THF-II-loti_TL_. RBS is highlighted in purple. (**B**) Structure architecture of RBS helix. RBS is highlighted in purple. (**C**) Base pairing between G11 and G51. N7 and O6 (carbonyl) of G11 each form one hydrogen bond with N2 (amino) and N1 (imino) of G51, respectively. (**D**) Base pairing between G12 and A50. O6 (carbonyl) and N1 (imino) of G12 each form one hydrogen bond with N6 (amino) and N1 of A50, respectively. (**E**) Base pairing between U13 and G49. O2 (carbonyl) and N3 (imino) of U8 each form one hydrogen bond with N1 (imino) and O6 (carbonyl) of G49, respectively.


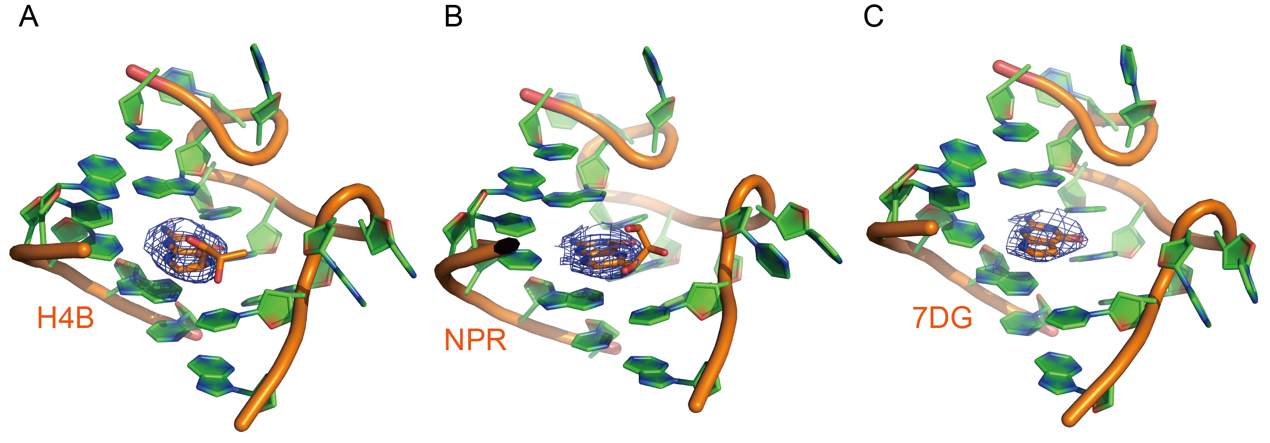


**Figure S3. Binding pocket architecture of THF-II-loti_TL_ bound with various analogs.** (**A-C**) Binding pocket architecture of THF-II-loti_TL_ bound with H4B (A), NPR (B) and 7DG (C) in cartoon representation. H4B, NPR and 7DG are highlighted in stick representation. The composite omit maps (contoured at 1.0 σ level) of the ligands are shown in tv-blue mesh.

**Supplementary Table S1. Thermodynamic parameters of ligand binding to THF-II riboswitch determined by ITC.**

| RNA | Ligand | MgCl_2_  (mM) | ΔH  (kcal/mol) | -TΔS  (kcal/mol) | ΔG  (kcal/mol) | N | K*_d_*  (µM) |
| --- | --- | --- | --- | --- | --- | --- | --- |
| THF-II-loti_62_ | THF | 0 | n.d. | n.d. | n.d. | n.d. | n.d. |
| THF-II-loti_62_ | THF | 0.5 | -12.4 | 6.94 | -5.49 | 1.2 | 93.9 |
| THF-II-loti_62_ | THF | 1 | -9.89 | 3.9 | -5.99 | 1.0 | 41.0 |
| THF-II-loti_62_ | THF | 2 | -15.8 | 9.77 | -6.01 | 1.1 | 39.2 |
| THF-II-loti_62_ | THF | 3 | -18.6 | 12.5 | -6.11 | 1.1 | 33.3 |
| THF-II-loti_62_ | THF | 5 | -16.8 | 10.7 | -6.14 | 1.0 | 31.7 |
| THF-II-loti_62_ | THF | 10 | -18.6 | 12.4 | -6.25 | 0.9 | 26.3 |
| THF-II-loti_62_ | NPR | 10 | -14.9 | 8.6 | -6.32 | 1.2 | 23.6 |
| THF-II-loti_62_ | H4B | 10 | -14.9 | 8.6 | -6.28 | 1.2 | 25.0 |
| THF-II-loti_62_ | 7DG | 10 | -17 | 10.9 | -6.04 | 1.0 | 37.6 |
| THF-II-loti_62_ | Folinic acid | 10 | n.d. | n.d. | n.d. | n.d. | n.d. |
| THF-II-loti_62_ | 5-methyl-THF | 10 | n.d. | n.d. | n.d. | n.d. | n.d. |
| THF-II-loti_62_ | 6-Biopterin | 10 | n.d. | n.d. | n.d. | n.d. | n.d. |
| THF-II-loti_TL_ | THF | 10 | -14 | 7.75 | -6.28 | 1.2 | 25.0 |
| THF-II-loti_TL_ -U44C | THF | 10 | n.d. | n.d. | n.d. | n.d. | n.d. |
| THF-II-loti_TL_ -U44A | THF | 10 | n.d. | n.d. | n.d. | n.d. | n.d. |
| THF-II-loti_TL_ -C22U | THF | 10 | n.d. | n.d. | n.d. | n.d. | n.d. |
| THF-II-loti_TL_ -C22G | THF | 10 | n.d. | n.d. | n.d. | n.d. | n.d. |
| THF-II-loti_TL_-M1 | THF | 10 | -23.1 | 17.4 | -5.69 | 0.9 | 67.2 |

n.d. here means not detected.

**Supplementary Table S2. Crystallographic statistics of THF-II-loti_TL_ in complex with ligands.**

| **THF-II-loti_TL_** | **THF-bound, Se-Urea-soaked** | **THF-bound** | **H4B-bound** | **NPR-bound** | **7DG-bound** | **C22G** |
| --- | --- | --- | --- | --- | --- | --- |
| **PDB code** | **7WI9** | **7WIB** | **7WIF** | **7WII** | **7WIE** | **7WIA** |
| **Data collection** |  |  |  |  |  |  |
| Wavelength (Å) | 0.979 | 0.979 | 0.979 | 0.979 | 0.979 | 0.979 |
| Resolution (Å) | 50-2.98  (3.09-2.98) | 50-2.85  (2.90-2.85) | 50-2.86  (2.96-2.86) | 50-2.75  (2.85-2.75) | 50-2.90  (2.95-2.90) | 50-3.22  (3.34-3.22) |
| Space group | *P* 3_1_21 | *P* 3_1_21 | *P* 3_1_21 | *P* 3_1_21 | *P* 3_1_21 | *P* 3_1_21 |
| Cell dimensions | 66.6 66.6 92.5 90.0 90.0 120.0 | 66.6 66.6 90.0 90.0 90.0 120.0 | 66.2 66.2 92.8 90.0 90.0 120.0 | 66.6 66.6 93.1 90.0 90.0 120.0 | 66.9 66.9 94.6 90.0 90.0 120.0 | 66.4 66.4 93.4 90.0 90.0 120.0 |
| Unique reflections | 5136 | 5768 | 5752 | 6536 | 6332 | 4048 |
| Completeness (%) | 99.8 (99.4) | 99.8 (100.0) | 100.0 (100.0) | 100.0 (100.0) | 98.83 (92.94) | 99.8 (100.0) |
| Rmerge (%) | 10.5 (119.5) | 10.9 (85.3) | 9.1 (57.1) | 9.2 (94.9) | 9.1 (115.2) | 21.0 (74.6) |
| Rpim (%) | 2.5 (27.9) | 3.4 (20.6) | 2.6 (13.4) | 2.5 (21.6) | 2.4 (28.4) | 5.2 (18.5) |
| CC1/2 (%) | 100.0 (93.5) | 93.8 (94.6) | 97.3 (98.0) | 98.9 (92.8) | 101.5 (87.3) | 97.6 (95.9) |
| Redundancy | 18.9 (19.0) | 14.7 (17.0) | 14.8 (18.8) | 17.0 (19.7) | 17.0 (15.4) | 17.8 (15.7) |
| *I*/σ(*I*) | 19.3 (2.2) | 22.9 (4.6) | 26.2 (7) | 27.1 (4.3) | 30.5 (2.5) | 15.5 (5.1) |
| **Refinement** |  |  |  |  |  |  |
| Rwork (%) | 20.2 (35.2) | 20.6 (40.8) | 19.9 (34.4) | 21.3 (47.4) | 18.2 (46.5) | 16.0 (22.2) |
| Rfree (%) | 22.8 (34.8) | 23.5 (36.8) | 20.9 (37.8) | 24.8 (51.0) | 21.6 (57.6) | 21.1 (23.3) |
| **R.m.s.d.** |  |  |  |  |  |  |
| Bond length (Å) | 1.25 | 1.36 | 1.17 | 1.08 | 1.30 | 1.49 |
| Bond angles (°) | 0.007 | 0.010 | 0.005 | 0.005 | 0.007 | 0.008 |
| **No. of atoms** |  |  |  |  |  |  |
| RNA | 1074 | 1074 | 1074 | 1074 | 1074 | 1078 |
| Ligand | 29 | 13 | 17 | 18 | 11 | - |
| **Average B factors (Å^2^)** |  |  |  |  |  |  |
| RNA | 95.5 | 98.9 | 86.7 | 90.0 | 107.1 | 114.5 |
| Ligand | 85.8 | 82.6 | 73.1 | 87.2 | 89.3 | - |

**Supplementary Table S3. Basic structural parameters for THF-II-loti_62_ RNA at various Mg^2+^ and ligand concentrations by SAXS.**

| RNA (µM) | Mg^2+^ (mM) | THF (mM) | ^a^*R_g_* (Å) | ^b^*R_g_* (Å) | *D_max_* (Å) | ^c^MW (kDa) | ^d^MW (kDa) |
| --- | --- | --- | --- | --- | --- | --- | --- |
| 30 | 0 | 0 | 25.6 ± 0.5 | 27.0 ± 0.06 | 97 | 20.1 | 19.5 |
| 30 | 5 | 0 | 24.5 ± 0.3 | 25.8 ± 0.04 | 92 | 19.4 |  |
| 30 | 5 | 0.3 | 24.0 ± 0.3 | 25.4 ± 0.04 | 88 | 19.9 |  |

^a^derived from Guinier fitting;

^b^derived from GNOM analysis;

^c^MW: molecular weight calculated based on the power law of volume of correlation;

^d^MW: molecular weight predicted from RNA sequence.
